# Biochemical changes and treatment in glaucoma

**Published:** 2015

**Authors:** IM Ciotu, I Stoian, L Gaman, MV Popescu, V Atanasiu

**Affiliations:** * Biochemistry Department, “Carol Davila” University of Medicine and Pharmacy, Bucharest, Romania; ** Department of Ophthalmology, Emergency Hospital Bucharest, Romania

**Keywords:** glaucoma, oxidative stress, neuroprotection, nitric oxide

## Abstract

Glaucoma is the second cause of blindness worldwide. This disease is a neurodegenerative disorder characterized by high intraocular pressure, loss of retinal ganglion cells (apoptosis). Even though there is much research done in this field, the results have not yet managed to stop the progression of glaucoma or to heal this pathology. Free oxygen radicals play a major role; they are formed in the aqueous humor and in the vitreous and they produce apoptosis of the neurons in the optic nerve head, degradation of the trabecular meshwork cells. The purpose of the article is to help in trying to understand the physiopathology of glaucoma and the efficacy of its treatments.

**Abbreviations:** IOP = intraocular pressure, NO = nitric oxide, NOS = nitric oxide synthase, ROCK = coiled coil-forming protein kinase, RCG = retinal ganglion cells, NGF = nerve growth factor.

## Introduction

Glaucoma represents the second cause of blindness in the world [**1**,**2**]. The estimated number of patients suffering from this disease is of about 68 million, of whom 6,7 million are blind [**3**].

In Romania, this pathology affects up to 160 000 people and the disease is more frequent in certain categories:

• over 40 years old (over 10% of all people aged 80 or more)

• have a first degree relative diagnosed with glaucoma

• have high IOP

• have high myopia

• are treated with corticosteroids 

• have high blood pressure [**4**]

However, almost 50% of the glaucoma patients remain undiagnosed.

The disease is classified in chronic open-angle glaucoma and chronic closed angle glaucoma, which in turn have other subtypes. Open angle glaucoma is the most frequent type, found in almost 50% of the patients [**5**].

**Etiology**

It was believed that glaucoma is the result of high intraocular pressure, but the latest studies showed that it is a degenerative optical neuropathy. Intraocular hypertension correlates with the visual field defects and loss of nerve fibers as seen on Optical coherence tomography (OCT) [**6**], but also with the perfusion of the head of the optic [**7**] nerve and the thickness of the cornea [**8**].

**Physiology**

The aqueous humor is produced by the ciliary processes in the posterior chamber and passes through the pupil in the anterior chamber [**9**]. From there, the aqueous humor exits the eye following two ways:

1. The trabecular meshwork - the main route of outflow (almost 90%) - it passes into Schlemm’s canal and after that, exits the eye through the episcleral veins [**10**,**11**].

2. The uveoscleral route - the aqueous humor passes on the anterior surface of the ciliary body in the suprachoroidal space and by way of the venous circulation of the ciliary body in the choroidal vessels and after that the scleral veins [**12**].

Intraocular pressure is mainly determined by the coupling of the production of the aqueous humor and the drainage of it, mainly through the trabecular meshwork located in the anterior chamber angle. Intraocular pressure varies throughout the night and day. IOP ranges between 10 and 21 mm Hg with a mean of about 15 or 16 mm Hg. The diurnal variation for normal eyes is between 3 and 6 mmHg, this may change with the arterial tension, breathing and fluid intake. During the night, intraocular pressure may not decrease.

**Oxidative stress in glaucoma**

Although the free radicals are quite well studied, their effects in the aqueous humor are incompletely understood.

The perturbation of the balance pre-oxidant/ antioxidant can lead to an increased oxidative damage [**13**]. Chronic changes (free radicals and reactive oxygen species) in the composition of the aqueous humor may induce the alteration (apoptosis) in the trabecular meshwork cells and in the head of the optic nerve. It is likely that glaucomatous patients might have a genetic predisposition, being more susceptible to reactive oxygen species.

**Nitric oxide (NO)**

This is a free radical which is formed from l-arginine by the enzyme NO synthetase (NOS). NOS has three isoforms: NOS-1 (this enzyme is associated with diminished bundles at the prelaminar region of the lamina cribrosa in glaucoma patients), NOS-2 (is associated with elevated IOP – the presence of this enzyme has a genetic predisposition), NOS-3 (vasodilator found in the prelaminar region of the optic nerve) [**14**,**15**]. It was shown that NOS-2 is absent in healthy patients, while NOS-1 and NOS-3 are upregulated in glaucoma patients. Studies showed that increased NO in the retina produces ischemia, inflammation and excitotoxicity [**16**]. NO has the ability to pass from one neuron to another bypassing synapses. The NO is a free radical with a moderate activity, the major problem being its growing effect on the other free radicals, which lead to retinal ganglion cells (RGC) apoptosis [**39**].

**Glaucoma treatment**

At present, there is an increasing need of new discoveries regarding both the medical and surgical treatment of glaucoma. Current treatment modalities are based on lowering the IOP, which is only one of the etiologic factors of the disease [**17**,**18**]. Recently, new therapies that lower IOP (by using Rho kinase inhibitors) have been developed, new surgical techniques that are less traumatizing and other therapies that act as neuroprotectors [**19**,**20**].

**Medical treatment**

Rho kinase inhibitors are a group of guanosine triphosphatases (GTPases). This group has a very important role in the activity of the cytoskeleton and the contraction and motility of the cell. Rho associated with coiled coil-forming protein kinase (ROCK) [**21**]. The hypothesis is that ROCK inhibitors may augment aqueous drainage; the inhibitors are targeting the actin cytoskeleton and cellular motility in the trabecular meshwork, Schlemm’s canal and ciliary muscle. The effect of the new class of ocular antihypertensive lowers the IOP by decreasing the resistance to aqueous outflow by cellular relaxation in the trabecular meshwork and Schlemm’s canal. For the moment, there are 2 drugs in stage 2 trials.

**Neuroprotection, neuromodulation and neurorecovery**

Other than the high IOP, glaucoma is characterized by RGC apoptosis, being also considered a degenerative disorder. The clinical trials focus on the prevention of the apoptosis of the RGC (neuroprotection), delaying the apoptosis of the RGC (neuromodulation) or reversing the process (neuro-recovery) [**22**,**23**].

The substances recently studied are the following:

• Memantine is an N-methyl-D-aspartate (NMDA) receptor antagonist. The results from the animal trials were statistically significant but efficacy in humans was not proven [**24**,**25**].

• Nerf growth factor (the group of endogenous proteins that controls the growth, division, maturation and proliferation of various cells). Lambiase and colleagues studied the intravitreal administration of NGF on experimental models of RGC degeneration [**26**]. The ability of the growth factor to penetrate the retina was impressive (this protein has a big molecular weight) [**22**]. Lambiase and colleagues treated 3 patients with advanced glaucoma with a murine topical NGF for a period of 3 months and the results showed an improvement in the visual acuity, contrast sensitivity, perimetry, and electrophysiological functions. No conclusions could be drawn from this small, short-term, open-label clinical study.

• Calcium Blocker channels (Nifedipine, Flunarizide)

• Inhibitors of glutamate release [**26**]

• Brimonidne [**27**]

• Antioxidants [**28**]

• Cannabinoids [**29**]

• Nitric Oxide Synthase (NOS-2) Inhibitors (Aminoguanidine)

• Gingko Biloba extract (GBE) (improves ocular blood flow) [**30**] used color Doppler imaging to measure ocular blood flow before the administration of GBE and after, noticing that it did not alter the arterial blood pressure, IOP or hart rate, but increased diastolic velocity in the ophthalmic artery.

• 17 beta-estradiol helps increase the viability, differentiation and survival of primary neuronal cultures in different parts of the brain. Three synthetic estrogen analogues (ZYC-1, ZYC-3, and ZYC-10) showed their efficacy of neuroprotection against glutamate-induced RGC cell death [**31**]. These results support the hypothesis that estrogen analogues may be useful in the neuroprotection of the retinal ganglion cells in ocular pathologies such as glaucoma [**32**].

• Erythropoietin [**33**,**34**] made a trial on a mouse model of glaucoma; in this trial, erythropoietin promoted RGC survival. This led to the suggestion that erythropoietin may have a potential therapeutic role as a neuroprotector in glaucoma [**23**,****35****].

o The most recently studied theories are the following:

• Neutrophine [**36**]

• Immunomodulatory Compounds

• Glatiramer Acetate (this medication is currently used for neuroprotection in multiple sclerosis)

• TNF-alfa blockers [**37**,**38**]

• Gene therapy

• siRNA

• stem cells

**Surgical treatment**

As time goes by, more and more surgical devices become available, with better results. There are devices that aim to improve the way the trabeculectomy works and the time the trabecular is functional [**22**].

• Ologen implant (a lyophilized porcine collagen matrix implant that is biodegradable) is used to provide a scaffold for fibroblast growth [**39**].

• Ex-press mini shunt- it is a stainless steel implant used under the scleral flap. Results are similar with the trabeculectomy procedure.

Devices that have the purpose of improving the fluid outflow into the Schlemm’s canal:

• The trabectome is an electrocautery device, used in the same time with the surgical procedure. The device makes a communication between the anterior chamber and Schlemm’s canal; the results are very good (lowering IOP by 40%) only if the operation is done as a standalone procedure.

• Glaukos istent is a titanium implant inserted through the trabecular meshwork. The benefit in lowering the IOP is modest in patients with open-angle glaucoma and cataract. The results are better in patients with secondary glaucoma.

Devices that have new ways of eliminating aqueous humor from the eye:

• CyPass micro-shunt: this shunt is placed into the suprachoroidal space and facilitates the outflow of the intraocular fluid into the uveoscleral pathway.

Devices that suppress the production of aqueous humor

• EyeOP1 this device produces external ultrasounds that destroy the ciliary body.

## Discussion

Nowadays, the glaucoma treatment is mostly based on the lowering of the IOP, the recent research showed that glaucoma is a multifactorial disease, which gathers immunologic changes, neurotrophic factor deficiency, glutamate-mediated excitotoxicity, immune-related phenomena, weak collagenous support at the lamina cribrosa, intracellular calcium influx, and free radical damage.

Although the mainstay treatment of glaucoma will remain the IOP lowering drugs, increased efforts will be made in finding alternative treatments. Neuroprotection seems to be a promising modality of slowing down or even stopping the course of the disease.

**Acknowledgement**

This paper is partly supported by the Sectorial Operational Programme Human Resources Development (SOPHRD), financed by European Social Found and the Romanian Government under the contract number POSDRU/159/1.5/S/137390.
